# Fas/FasL pathway and cytokines in keratinocytes in atopic dermatitis – Manipulation by the electromagnetic field

**DOI:** 10.1371/journal.pone.0205103

**Published:** 2018-10-04

**Authors:** Lukasz Szymanski, Aleksandra Cios, Sławomir Lewicki, Pawel Szymanski, Wanda Stankiewicz

**Affiliations:** 1 Department of Microwave Safety, Military Institute of Hygiene and Epidemiology, Warsaw, Poland; 2 Department of Regenerative Medicine and Cell Biology, Military Institute of Hygiene and Epidemiology, Warsaw, Poland; Universidad Pablo de Olavide, SPAIN

## Abstract

**Background:**

Atopic dermatitis (AD) is one of the most frequent skin diseases. Changes of the keratinocytes functionality play a major role in the development of AD. For example, activation of the Fas (CD95)/FasL (CD178) pathway in AD does not lead to extensive apoptosis in skin. Binding of the Fas receptor to its protein ligand—FasL, which are present on the (AD)-modified keratinocytes, should result in the sequential induction of cell death, but there is no evidence of extensive apoptosis of these cells. This suggests that non-apoptotic mechanism of Fas/FasL pathway is commonly encountered, although not examined in the case of AD, phenomenon. An electromagnetic field, which was used to influence cultured cells in this study, can modulate proliferation, apoptosis, differentiation, and metabolism in various cells.

**Objective:**

Here, we evaluate the possibility to manipulate the immune activation of AD keratinocytes and their response to the electromagnetic field, which was not tested before.

**Methods:**

Keratinocytes isolated from the skin of healthy subjects (n = 20) and patients with atopic dermatitis (n = 20) as well as HaCaT and PCS-200-010 cell were exposed to the 900 MHz electromagnetic field for 60 minutes. Cytometric analysis of viability, Fas/FasL, p-ERK, p-p38 and p-JNK expression and Luminex analysis of cytokine concentration were performed in two-time points: 4 and 24 hours after the exposition.

**Results:**

This research has shown upregulated Fas, FasL, p-ERK, p-p38, and p-JNK expression along with increased cytokine secretion (IL-1β, IL-4, IL-8, IL-10, IL-12p70, IL-13, IL-17A, IL-31 and TNFα) by keratinocytes derived from the skin of patients with the AD when compared with healthy control. Exposure of keratinocyte cultures obtained from AD patients to EMF resulted in a decrease of 1β, IL-4, IL-10, IL-12, I L-13, IL-17, IL-31 and TNFα levels. Keratinocytes derived from the skin of AD patients are characterized by elevated Fas and FasL expression when compared to healthy control.

**Conclusion:**

Apoptotic and nonapoptotic activation of the Fas/FasL-dependent signaling pathway may play a significant role in the pathogenesis of AD, by adjusting the local cytokine and chemokine environment at the site of inflammation. Moreover, the electromagnetic field exhibits strong immunomodulatory effects on AD-modified keratinocytes.

## Introduction

One of the most frequent skin disorder is atopic dermatitis (AD) which is characterized by a disturbance of epidermal barrier function leading to dry skin and chronic relapsing form of skin inflammation. Another important mechanism mentioned by Bien. et. al. is keratinocyte apoptosis-mechanism of eczema and spongiosis formation, mostly seen in acute and subacute lesions. Several factors play important role in the pathogenesis of AD which involve genetic, environmental, skin barrier, psychological and immunological factors. In various studies, Fas/FasL mediated keratinocyte apoptosis was demonstrated to be an important component of eczema and spongiosis in AD patients[1]. Epidermal keratinocytes express Fas and FasL proteins in low amounts [2]. Abnormal expression of lytically active FasL was found in inflammatory skin diseases such as toxic epidermal necrolysis, atopic dermatitis and allergic contact dermatitis [3]. As described in detail previously [1], scientific evidence indicates that Fas/FasL death receptors activate inflammatory or proliferative signaling via NF-κB or MAP kinase pathway [4]. MAPK pathway consists of three major components: proline-directed serine/threonine kinase known as the extracellular signal-regulated kinase (ERK), p38 MAPK and c-Jun NH_2_-terminal kinase (JNK). MAPK pathway and these kinases are activated by various stimuli and play pivotal roles in processes such as apoptosis, cellular survival, proliferation and cytokines expression [5,6]. Moreover, in AD skin FAS-receptors are expressed, but there is no evidence of extensive apoptosis of these cells suggesting that non-apoptotic mechanism of Fas/FasL pathway is commonly encountered, although not examined in the case of atopic dermatitis, phenomenon. It was shown that FasL induces production of cytokines which trigger an inflammatory response in keratinocytes. This indicates the existence of an autoactivating loop of cytokines in the skin [7]. Finally, in 2006 Farley et al. demonstrated that FasL triggered an NF-kB-dependent mRNA accumulation of inflammatory cytokines and chemokines such as TNF-α, IL-6, and IL-1, CCL2, CXCL1, CXCL3 and CXCL8/IL-8, and the adhesion molecule ICAM- 1 in HaCaT cells and in the reconstructed human epidermis (RHE) [7].

Electromagnetic field (EMF) is a combination of an electric field and a magnetic field governed by Maxwell's equations. EMF is characterized by an amplitude of the electric or magnetic components, a frequency, and a wavelength. Exposure of cells to the electromagnetic field causes the activation of the sodium-potassium pump, NHE3 channel, AChR transport proteins (Acetylcholine receptor) and NMDAR (N-methyl-D-aspartate receptor), resulting in increased inflow of Na^+^ and Ca^2+^ into the cell [8]. Elevated concentrations of Na^+^ and Ca^2+^ causes depolarization and reorganization of the cytoskeleton [9]. Furthermore, the EMF may activate the EGFR in the ligand-independent manner, which leads to activation of MAPK and PI3K pathways and consequently to activation of the mTOR pathway [10,11]. These pathways regulate many important processes in the cell, therefore the cell stimulation by electromagnetic fields can lead to more intense apoptosis, increased cell proliferation, changed viability and cell differentiation. Finally, an exposure to EMF can inhibit the release of proinflammatory TNFα, IL-1, IL-6, IL-10 while stimulating the release of anti-inflammatory IL-10 [12].

We hypothesize that it is possible to alter the cytokine secretion and Fas/FasL expression in AD keratinocytes using EMF. Therefore, the purpose of this study was to analyze cytokine and chemokine secretion profile and Fas/FasL expression of active AD keratinocytes, as well as to evaluate effects of the EMF exposition in these cells. Moreover, the percentage of p-ERK, p-p38, and p-JNK positive cells were evaluated. Finally, this research was designed to evaluate the suitability of commercially available cell lines as a material for AD and EMF studies.

## Materials and methods

The research was approved by the local Ethics Committee associated with Military Institute of Aviation Medicine (decision number 2/2016). The material was isolated under signed conscious consent of the patient form. All research was performed in accordance with relevant guidelines and regulations.

### Cell lines

**Normal, primary human keratinocytes (control group)**–adherent cell isolated from the forearm epidermis of 20 single donors (n = 20). The inclusion criterion included the absence of cancer, systemic and chronic diseases and the lack of pharmacological treatment (at least one month before isolation of the cells) as well as the absence of aspecific inflammation and not excessive use of tobacco and alcohol. Cells at the second and third passage were used for experiments.

**AD-altered, primary human keratinocytes (study group)**–adherent cells isolated from the epidermis of 20 single donors (n = 20). Skin grafts were extracted from different locations on the donor body, depending on the location of the AD lesion. Only untreated patients who did not receive steroid or antibiotic medication with EASI score greater than 20 were included in the study. EASI score greater than 20 (on a 0–72 scale) indicates an acute AD [13]. Cells at the second and third passage were used for experiments.

The basic information about patients were collected in Table 1.

**Table 1:** Basic information about patients in control and study group.

	**Control group**			
	Female		Male	
	Average	Standard deviation	Average	Standard deviation
Age [years]	44	9,8	44,8	8
Weight [kg]	60,6	7,1	74,8	2,6
Height [cm]	165,6	2,3	173,7	4,9
BMI	22,41		24,49	
	**Study group**			
	Female		Male	
	Average	Standard deviation	Average	Standard deviation
Age [years]	42,8	9,3	46,5	6,5
Weight [kg]	61,4	6	77,1	6,7
Height [cm]	166,8	3,8	175,6	5,9
BMI	21,87		24,86	

**PCS-200-010** (ATCC)—Primary Epidermal Keratinocytes; Normal, Human, Neonatal. An adherent cell line from a single donor, obtained from a newborn's foreskin. Cell doubling time was around 26 hours.

**HaCaT**—adherent, a non-cancerous line of human keratinocytes. The cell line was immortalized by a spontaneous mutation leading to aneuploidy. HaCaT cells despite the aneuploidy are characterized by the standard features (structure and morphology, expression markers, ability to differentiate) for normal human keratinocytes [14]. HaCaT cell line was a gift from Independent Laboratory of Nanobiology and Biomaterials, Military Institute of Hygiene and Epidemiology, Warsaw, Poland.

Skin-derived cells were isolated using enzymatic digestion [15]. Briefly, adipose tissue was removed using scissors and the remaining tissue was incubated in the Dispase II solution (5 u/mL, ThermoFisher Scientific, Poland, Poland) for 60 min. Later the epidermal layer was removed and incubated with 1:1 trypsin-accutase solution (0,25% Trypsin-EDTA, StemPro Accutase, ThermoFisher Scientific, Poland) for 20 min. After that, Trypsin Neutralizer Solution (ThermoFisher Scientific, Poland) was added to inhibit enzymatic digestions before cells were centrifuged (500g, 5min) and seeded in the culture flask. Cells were cultured in standard conditions (37°C; 5% CO2) in EpiLife Medium (ThermoFisher Scientific, Poland) supplemented with Penicillin-Streptomycin (1000 u/mL and 10 mg/mL, respectively, ThermoFisher Scientific, Poland) and Human Keratinocyte Growth Supplement (ThermoFisher Scientific, Poland). After reaching about 85–90% confluency cells were transferred to the 6 well plates (3 x 10^5^ cells per well, 4 wells per sample) and incubated for 2 days. HaCaT and PCS-200-010 cells were also seeded at the density of 3 x 10^5^ cells per well, 4 wells per sample.

With a freshly changed culture media, one of the plates was exposed to EMF for 60 minutes in custom and validated anechoic chamber integrated with a CO_2_ incubator. The experiments were performed at least in three independent time points.

### Electromagnetic exposure system and conditions

**Fig 1:**
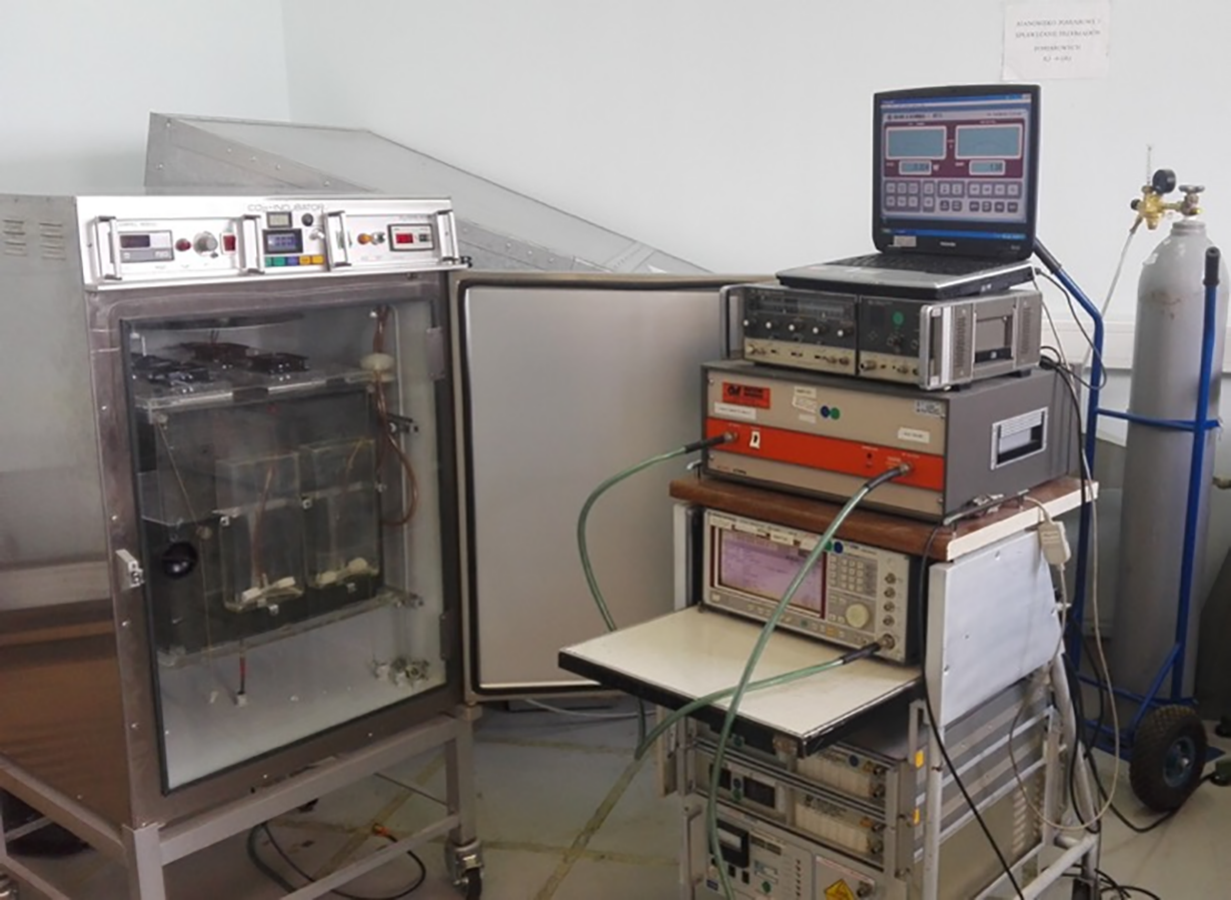
Custom electromagnetic field exposure system. The system consists of the electromagnetic generator, power amplifier, antenna, anechoic chamber and CO2 incubator.

Custom assembled EMF system consisting of the electromagnetic generator, power amplifier, antenna, anechoic chamber and CO_2_ incubator was used in experiments (Fig 1).

Temperature variation in the incubator and cell culture medium was validated. No changes in temperature were observed during 1-hour, 900 MHz exposition. What is more, homogeneity of the electromagnetic field and specific absorption rate (SAR) distribution was also validated [16].

Exposure conditions selected based on previous experiments performed in Department of Microwave Safety, Military Institute of Hygiene and Epidemiology [17–19], were as follows:

- Frequency F = 900 MHz
- Wavelength = 33 cm
- Effective electric field E = 20 V / m
- Ti pulse duration = 570 μs
- pulse repetition period Tp = 1.14 ms,
- SAR = 0.024 W / kg
- Time: 60 min

4 and 24 h after the EMF exposure the analyzes were performed. Atopic dermatitis is a type I IgE-mediated hypersensitivity reaction with an immediate reaction occurring after several minutes and a late reaction occurring between 6^th^ and 10^th^ hour after exposure to an allergen. However, it is known that the mast cells are mainly responsible for the immediate and late reaction. In this study, the response of keratinocytes was evaluated. Time points of 4h and 24h were selected because literature data indicate that after stimulation, secretion of preformed cytokines occurs between 2^nd^ and 4^th^ hour, and the secretion of de novo formed cytokines occurs between 20^th^ and 24^th^ hour after exposure [20,21]. What is more, a pilot kinetic study of cytokine secretion was performed allowing for the selection of time points.

### Luminex and cytometric analysis

Cell viability assays (rh AnnexinV-APC, 7-AAD Viability Staining Solution, Binding Buffer for Annexin V) was performed as previously described [22]. Fas (Anti-Human CD95 (DX2) FITC) and FasL (Anti-Human CD178 (CD95L) (NOK-1) PE) expression were analyzed by flow cytometry (FACS Calibur, BD, USA). Appropriate controls were performed before conducting the analysis. Cytokines and chemokine concentration analysis were performed using Luminex MagPIX platform and ProcartaPlex magnetic beads. The reagents were purchased from Affymetrix eBioscience, Austria.

MAPK pathway activation analysis was performed using flow cytometry (FACS Calibur, BD, USA) and phospho-specific antibodies (Phospho-ERK1/2 (Thr202, Tyr204) Monoclonal Antibody (MILAN8R), Phospho-p38 MAPK (Thr180, Tyr182) Monoclonal Antibody (4NIT4KK) and Phospho-SAPK/JNK (Thr183/Tyr185) (G9) Monoclonal Antibody). Results were validated with appropriate isotype controls.

Reactive oxygen species (ROS) generation was measured using FACS Calibur cytometer and CM-H2DCFDA General Oxidative Stress Indicator. Antibodies and CM-H2DCFDA were acquired from ThermoFisher Scientific, Poland.

### Statistical analysis

Evaluation of data distribution using the Shapiro-Wilk test showed a normal distribution of the obtained results except for the data from ROS analysis. Statistical evaluation of the results was performed using one-way analysis of variance (ANOVA) with post-hoc Tukey’s test for cytometric results and post-hoc Bonferroni’s test for data obtained with Luminex platform. ROS generation was evaluated using Mann-Whitney test. Results of Fas/FasL expression and cytokines secretion are presented as a median ± range. Cytometric results of a percent of cells with phosphorylated protein kinase (ERK, p38 or JNK) are presented as a mean ± SD. Results at p <0.05 were considered statistically significant. GraphPad Prism ver.7 software was used to perform statistical calculations (La Jolla, CA, USA).

## Results

**Fig 2:**
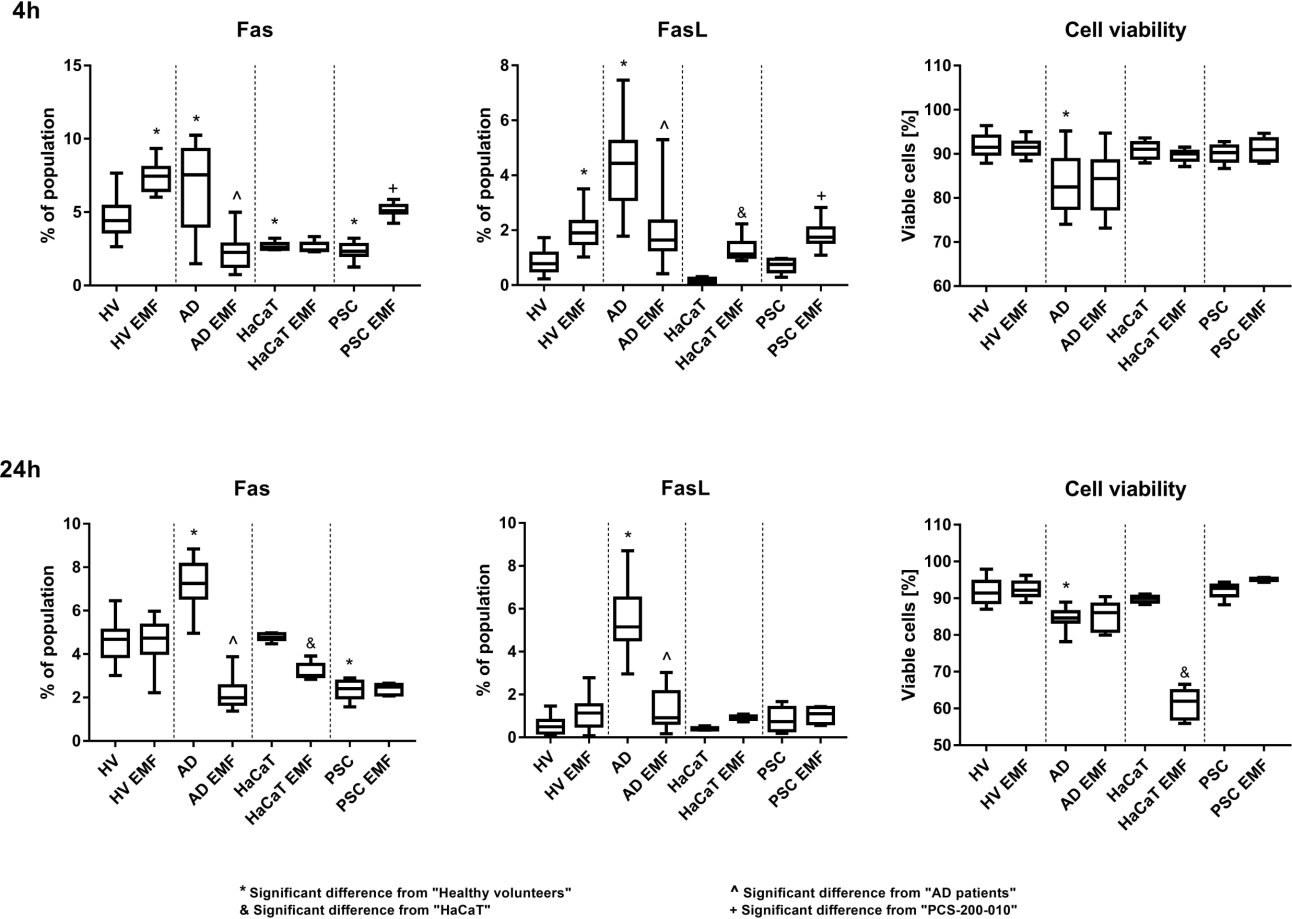
Fas, FasL expression and cell viability in keratinocytes. Results are presented as the median ± the range [%]. Data acquired by flow cytometry. n = 20 for primary cells and n = 10 for cell lines. Results were considered significant for p<0,05, (ANOVA with post-hoc Tukey’s test).

In this study, higher expression of Fas and FasL was observed in cells obtained from AD patients when compared to healthy volunteers (HV) (4^th^ and 24^th^h). Exposition to EMF resulted in increased Fas expression in cells from HV and PCS-200-010 cell line in the 4^th^ hour and decreased Fas expression in keratinocytes isolated from AD patients in the 4^th^ and 24^th^ hour of the experiment. HaCaT cells reacted differently to EMF exposition with decreased expression of Fas and reduced viability in the 24^th^h of the experiment. FasL expression increased significantly, 4h after the EMF exposition, in cells obtained from HV, PCS-200-010 and HaCaT cells, and decreased in the 4^th^ and 24^th^h of the study, in cells isolated from AD patients (Fig 2)

Keratinocytes isolated from AD patients exhibit increased secretion of IL-1β, IL-4, IL-8, IL-10, IL-12p70, IL-13, IL-17A, IL-31 and TNFα than cells obtained from HV. Only the cells isolated from AD patients were shown to have the ability to secrete IL-17A. HaCaT cells exhibit decreased secretion of IL-8, IL-10, IL-12p70 and IL-13, whereas PCS-200-010 cells were found to have highly increased secretion of IL-8.

**Fig 3:**
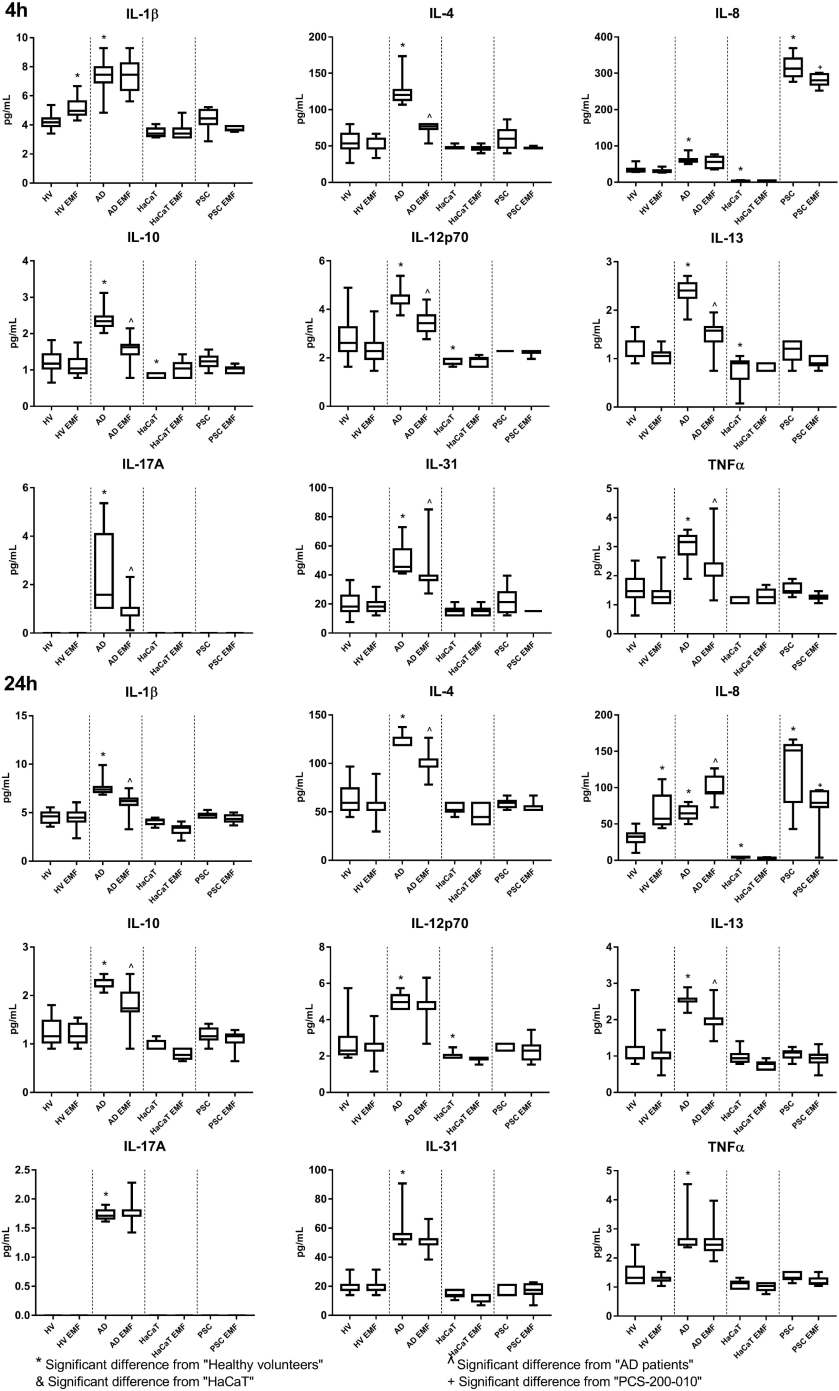
IL-1β, IL-4, IL-8, IL-10, IL-12p70, IL-13, IL-17A, IL-31 and TNFα concentration in keratinocytes. Results are presented as the median ± the range [pg/mL]. Data acquired using Luminex MagPix. n = 15 for primary cells and n = 10 for cell lines. Results were considered significant for p<0,05, (ANOVA with post-hoc Bonferroni’s test).

Exposure to 900MHz of AD keratinocytes resulted in near immediate and short term (<24h) decrease of IL-12p70, IL-17A, IL-31 and TNFα, and long term (>24h) decrease of IL-4, IL-10, IL-13 levels in the supernatant. In the 24^th^h of the study, exposure to EMF resulted in a decrease of the IL-1β mean concertation level in the AD derived cell culture supernatant and near immediate and short-term increase of IL-1β concentration in healthy cell culture supernatants. EMF exposure also increased secretion of IL-8 (in the 24^th^ hour of the study) by keratinocytes obtained from healthy volunteers and patients with the AD. On the contrary, exposition of the PCS-200-010 to the EMF resulted in a decrease of the IL-8 level in cell culture supernatants. No statistically significant differences were found in the supernatant concentrations of the investigated cytokines between exposed and unexposed HaCaT cells (Fig 3).

Phosphorylated-ERK (p-ERK) kinase was more abundantly expressed in cells obtained from AD patients when compared to HV (4^th^ and 24^th^h). HaCaT and PCS-200-010 cells were found to have fewer p-ERK positive cells than HV (4^th^ and 24^th^h). EMF exposure resulted in decreased number of p-ERK positive cells in AD derived keratinocytes (4^th^h) and increased in HaCaT cells (4^th^h).

P-p38 was found in a higher percentage in AD derived keratinocytes and a lower percentage of cells in HaCaT and PCS-200-010 when compared to HV (4^th^h and 24^th^h). Exposition to EMF resulted in decreased number of p-p38 positive cells in AD derived keratinocytes (4^th^h) and PCS-200-010 cells (4^th^ and 24^th^h).

**Fig 4:**
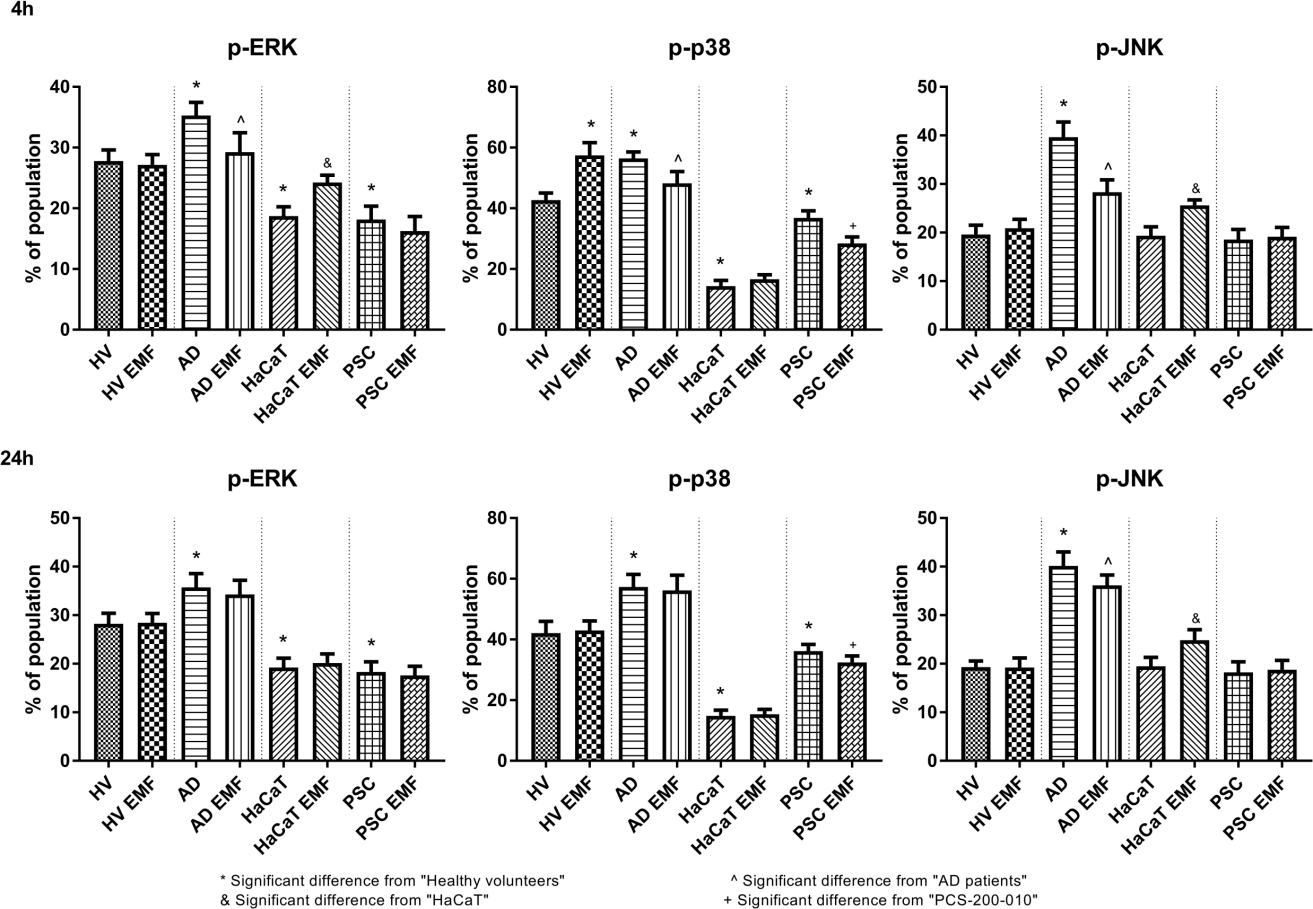
Percentage of p-ERK, p-JNK, p-p38 MAPK positive keratinocytes. Results are presented as the mean ± SD [%]. Data acquired by flow cytometry. n = 18. Results were considered significant for p<0,05, (ANOVA with post-hoc Tukey’s test).

P-JNK was found in a higher percentage in AD derived keratinocytes when compared to HV (4^th^h and 24^th^h). EMF exposure resulted in a decreased percentage of p-JNK positive cells in AD derived keratinocytes and increased in HaCaT cells (4^th^h and 24^th^h) (Fig 4).

In order to investigate increased apoptosis rate in HaCaT cells after EMF exposition, relative ROS generation was measured using flow cytometry. 60 min EMF exposure resulted in increased ROS production in HaCaT cells when compared to untreated control (2.426 vs 1 in 4^th^ h and 1,937 vs 1.042 in 24^th^ h, respectively).

## Discussion

Immunomodulating properties of the electromagnetic field can significantly affect the profile of chemokines and cytokines produced by keratinocytes, thus the recruitment of the different T-cell subpopulations. Furthermore, several studies have suggested Fas-mediated elimination of antigen-presenting cells as an important mechanism down-regulating the induction of autoimmune responses. Restricting DC priming functions through Fas–FasL interactions were shown to be a potent mechanism employed by CD4+/CD25+ regulatory T cells (Tregs) to restrict CD8+ T-cell-mediated allergic immune responses in the skin. Therefore, death receptors, such as Fas/FasL, may induce non-apoptotic signals and initiate cellular inflammatory responses rather than modulate apoptosis. Thus, Fas/FasL may shape the quality and quantity of the cytokine cocktail produced during the effector phase of the AD. These functions may have important and distinct pathophysiological consequences during different stages of AD inflammatory reaction.

In this study, we demonstrated that EMF downregulates the expression of Fas, FasL, percentage of p-ERK, p-JNK and p-p38 MAPK positive cells, and the secretion of IL-1β, IL-4, IL-10, IL-12p70, IL-13, IL-17A, IL-31 and TNFα in the AD keratinocytes, which should have a positive impact on the weakening of the symptoms. Although the molecular mechanism of EMF-Fas interaction is not yet known, it was shown that exposure of human endothelial cell line, EA.hy926 to the EMF caused the decrease in the expression of Fas mRNA [23]. Based on literature analysis, it is highly probable that the NF-κβ pathway and the MAPK-dependent pathway may be a potential site for regulation of the cytokine microenvironment in the AD that is affected by EMF. For example, FasL and TNF are functionally related molecules that initiate apoptosis. Although each of the molecules initiates apoptosis by another signaling pathway, it has been shown that FasL can activate the NF-κβ pathway, which is a regulator of TNFα expression. Activation of the Fas/FasL-dependent signaling pathway leads to stimulation of TNFα production [24]. Therefore, it is possible that reduction of Fas/FasL expression caused by EMF exposure may lead to inhibition of NF-κβ pathway and thus decreased TNFα secretion. What is more, TNFα secretion is also mediated through the ERK and p38 MAPK pathway [25], which were independently downregulated by EMF exposure. Altogether, the data suggest that EMF downregulates TNFα secretion via ERK and p38 MAPK pathways in AD derived keratinocytes. IL-31 secretion is also regulated by STAT6/NF-κβ signaling pathway and therefore, reduced expression of Fas may lead to reduced activation of NF-κβ, thereby resulting in decreased secretion of IL-31 [26].

What is more, AD derived keratinocytes are characterized by the higher percentage of p-ERK, p-p38, and p-JNK positive cells when compared with HV, HaCaT, and PCS-200-010 cells. Since, upregulated p38 and ERK are associated downregulated filaggrin and involucrin expression [27], p38 MAPK signaling pathway positively regulates the production of proinflammatory cytokines [28], and JNK is negatively associated with differentiation of epidermal keratinocytes [29], therefore the data obtained suggests possible molecular pathway responsible for AD symptoms manifestation. What is more, Sun et al. demonstrated that IL-1β production in keratinocytes is regulated via p38 [30], thus considering the results of this study (fewer p-p38 positive cells and decreased IL-1β secretion) it is possible that EMF regulates IL-1β production and secretion via p38 MAPK in AD keratinocytes.

Analogous studies were performed for HaCaT and PCS-200-010 cell lines. Our research partially confirms findings of Patruno et. al. in which they demonstrated upregulation of p-ERK, p-p38 and p-JNK in HaCaT cells after exposition to extremely low frequency electromagnetic field (ELF-EMF) [31]. In this research, we observed upregulation of p-ERK and p-JNK but not p-p38 MAPK in HaCaT cells after 60 minutes of 900 MHz EMF exposure. These results are in no way contradictory because effects of EMF exposure are both cell and frequency (wavelength) specific.

Because of different base expression of Fas/FasL and cytokines secretion, and different immune response of the PCS-200-010 and HaCaT cells to EMF exposure, those cell lines are not a suitable model for investigation of EMF exposition effects and can’t replace, primary human keratinocytes derived from the skin. Interestingly, decrease in HaCaT cell viability, after EMF exposure, is probably caused by activation of ERK, JNK MAP kinase and mTOR (PI3K / Akt) pathways, as well as, increased levels of reactive oxygen species that led to increased apoptosis of these cells.

The electromagnetic field may have therapeutic potential in skin diseases like atopic dermatitis however it also has limitations. Overexposure, different frequency or higher SAR may cause thermal effects that will be harmful to cells and organs [32]. Effects of EMF exposure are also cell type-dependent [33]. Moreover, Huang et al. observed that EMF may also differently affect the same type of cells i.e. epidermis keratinocytes [34]. We also confirmed this observation. Exposure to EMF results in reduced survival in HaCaT cells but not in primary dermal keratinocytes. Therefore, every potential therapeutic strategy involving the use of the EMF should be carefully studied in pre-clinical and clinical trials.

In conclusion, both apoptotic and nonapoptotic activation of the Fas/FasL-dependent signaling pathway may play a significant role in the pathogenesis of AD, by adjusting the local cytokine and chemokine environment at the site of inflammation. Moreover, to the best of our knowledge, this is the first publication to present that the electromagnetic field exhibits strong immunomodulatory effects on AD-modified keratinocytes. Finally, obtained data suggests that MAPK pathways mediated by ERK, JNK, and p38 protein kinases are responsible for AD symptoms manifestation. These findings suggest that EMF may be potentially used as adjuvant therapy in atopic dermatitis, however before human treatment its biological potential should be evaluated in pre-clinical studies.
